# COVID-19 and ethics in action: Insights from African research committees

**DOI:** 10.4102/jphia.v17i1.1540

**Published:** 2026-02-23

**Authors:** Alemseged Abdissa, Solomon M. Abay, Akililu Alemu Ashuro, Derbew Fikadu Berhe, Tseday Tilahun Degafa, Nchangwi Syntia Munung, Godfrey B. Tangwa, Juntra Karbwang, Yimtubezinash Woldeamanuel

**Affiliations:** 1 Armauer Hansen Research Institute, Addis Ababa, Ethiopia; 2 College of Health Sciences, Addis Ababa University, Addis Ababa, Ethiopia; 3 University of Global Health Equity, Kigali, Rwanda; 4 Department of Medicine, University of Cape Town, Cape Town, South Africa; 5 Cameroon Bioethics Initiative, Yaounde, Cameroon; 6 University of Yaounde 1, Yaounde, Cameroon; 7 Institute of Tropical Medicine, Nagasaki University, Nagasaki, Japan

**Keywords:** research ethics committee, Africa, COVID-19, PABIN, SIDCER

## Abstract

**Background:**

Research ethics committees (RECs) in Africa face challenges, including inadequate institutional support, low member engagement, and limited ethical review capacity. The COVID-19 pandemic added another layer of pressure on RECs.

**Aim:**

To delineate the activities of African RECs and pinpoint challenges encountered during the COVID-19 pandemic.

**Setting:**

The study was conducted across multiple RECs in various African countries.

**Methods:**

This cross-sectional study was conducted across multiple African countries to provide an overview of the functioning of RECs during the COVID-19 pandemic.

**Results:**

Chairs reported a substantial increase in protocol reviews, from 5860 in 2019 to a 12% (*n* = 744) increment in 2020. Amid the pandemic, there was a noticeable rise in research protocol amendments (79%, *n* = 38) and deviations. The vast majority of RECs (96%, *n* = 46) adhered to COVID-19 prevention institutional policies and limited face-to-face meetings. Challenges encountered in reviewing COVID-19-related proposals were linked to risk and/or benefit assessments and scientific designs.

**Conclusion:**

The study underscores the impact of the COVID-19 pandemic on REC functioning in Africa, marked by a surge in proposal volumes and the emergence of new ethical challenges. To address these challenges, there is a pressing need to nurture RECs in the region through diverse strategies, including capacity-building initiatives such as the Strategic Initiative for Developing Capacity in Ethical Review (SIDCER) recognition programme. Additionally, establishing periodic training opportunities through suitable platforms can further enhance the resilience and effectiveness of RECs.

**Contribution:**

This study contributes to understanding how RECs in African countries adapted their review processes during the COVID-19 pandemic, highlighting the need for establishing periodic training opportunities through suitable platforms.

## Introduction

Severe acute respiratory syndrome coronavirus 2 (SARS-CoV-2), the causative pathogen for COVID-19, first emerged in Wuhan, China, in December 2019. Globally, COVID-19 has caused several serious challenges, including significant lifestyle disruptions, economic crisis and increased levels of anxiety and fear in communities regarding disease management and infection spread.^1,2^ As the virus is highly transmissible and has a pathogenic feature, managing the infection requires a special healthcare setup and new management modalities that are designed to contain the virus and provide services for infected individuals.^3^ Since the pandemic emerged, the global scientific community has been racing to define the virus and find potential preventive and or curative remedies.^2,4^ In the early phase of the pandemic, research ethics was a very sensitive issue because of the poorly understood nature of the coronavirus and the lack of proven effective treatment for COVID-19.^5^ This has resulted in a significant burden and challenge to the research ethics committees (RECs). During the pandemic, RECs have faced multiple challenges, such as limited institutional support, conflicts of interest and limited capacity to conduct the review, a lack of specific guidelines and standards for protocol review in the early phase of the pandemic to handle COVID-19-related proposals, pressure from the scientific community to speed up the protocol review process and COVID-19-related restrictions for REC meeting.^5,6,7^

COVID-19-imposed RECs challenges were more pronounced in low- and middle-income countries, including those in Africa. One of the most important challenges was the lack of appropriate knowledge and experience in reviewing interventional protocols, mainly clinical trials.^8,9,10^ Globally, the lowest numbers of clinical trials are reported from Africa, and this may result in limited experience in reviewing COVID-19 clinical trials in the region.^11,12^ Because of restrictions and poor Internet connectivity, REC members in Africa may not get training and updated COVID-19 information. Moreover, RECs face difficulty meeting virtually and reviewing protocols online.^13^ Although there are no studies on the psychological impact of COVID-19 on REC members, it is expected that stress, fear and uncertainty might affect RECs activities. Another challenge for African RECs could be institution-related. Most African RECs lack websites and updated member databases, hindering virtual access and survey distribution.^14,15^

Studies on African REC challenges and experiences during public health emergencies (PHE) are limited.^16,17^ In addition, most of the RECs had no standard operating procedures (SOPs) to facilitate the rapid review of research in PHE.^13^ In 2000, the World Health Organization’s Special Programme for Research and Training in Tropical Diseases (WHO-TDR) spearheaded the establishment of REC networks as part of the Strategic Initiative for Developing Capacity in Ethics Review (SIDCER). Now, after two decades, two regional networks continue to operate: the Forum of Ethical Review Committee in Asia and the Pacific (FERCAP) and the Pan African Bioethics Initiative (PABIN) in Africa. This study, conducted by the PABIN, aimed to delineate the activities of African RECs and pinpoint challenges and capacity-building gaps encountered during the COVID-19 pandemic. Findings from this study offer recommendations to strengthen the resilience and preparedness of RECs for future pandemics, thereby improving their performance.

## Research methods and design

### Study design

This cross-sectional study, spanning multiple countries in Africa, was carried out collaboratively by the PABIN in conjunction with the Forum of Ethical Review Committee in Asia and the FERCAP and the World Health Organization’s Special Programme for Research and Training in Tropical Diseases (TDR).

### Recruitment strategy of research ethics committees

The investigators leveraged their network to engage researchers across various African countries, compiling a comprehensive list of contact addresses for RECs. To enhance participant outreach, we systematically explored the websites of African academic and research institutions. A purposive sample of 250 REC members was specifically targeted for this study. A total of 600 emails, each containing the Google form link, were dispatched to REC chairs, with a request to disseminate the link among their respective members. Following the initial distribution of the online survey, email reminders were sent up to five times to prompt responses, ultimately yielding 98 (49%) participants who responded to our inquiry.

### Data collection

Data collection instruments were prepared in English and translated into French for French-speaking countries. A small-scale pretest was done at local RECs to ensure the validity and reliability of the data collection instruments. Data were collected from July 2021 to September 2021 using an online Google form. Information collected includes REC characteristics, activities and working documents; the number of protocols initially reviewed in 2019 and 2020 during the COVID-19 outbreak; types of protocols reviewed, membership profile, number of COVID-19 protocols reviewed, common review issues found in COVID-19 protocols and training needs. Most of the questions are similar for both groups, but a few questions are different and completed by the chairperson and members based on their roles and responsibilities. Knowledge and attitude questions were initially prepared on five Likert scales. For an easy description of the data, we dichotomised knowledge questions into those with correct answers and those with incorrect answers, presenting the proportions of respondents with correct answers. For the attitude question type, the five-point Likert scale (strongly disagree, disagree, neutral, agree, strongly agree) was converted to three-point Likert scale: (strongly) disagree, neutral and (strongly) agree.

### Data collection process

After getting the REC approval, African RECs were contacted, and data collection was initiated using a Google Form by reaching out to REC chairs. The study had two sets of questionnaires: questionnaires to be filled by REC chairs and questionnaires to be filled by members. Chairs answered a form describing REC characteristics and activities during the COVID-19 outbreak. RECs members provided information on committee membership profiles, protocols reviewed, challenges encountered, training needs, and their knowledge and attitudes about the review of COVID-19 related research.

### Data management and analysis

Google Form data were exported as Excel spreadsheets, and Statistical Package for Social Sciences (SPSS) version 25 was used for data processing and analysis. Because of the nature of the data, we used descriptive statistics to explore REC characteristics, functioning and challenges during the pandemic. ArcGIS software was also used to locate the geographical location of REC chairs and members in Africa.

### Operational definition of variables

Exemption of protocols from ethics review: an ethics approval application that is not subject to expedited or full board review.

Expedited review: when the research procedures present no more than minimal harm to the research participants or communities.

Full board review: research involves more than minimal risk to human subjects or has been referred to the committee by an expedited reviewer or the Chair.

Joint or centralised review of multisite protocols is a process that involves the use of a single REC to review and approve research protocols for multiple sites.

### Ethical considerations

Ethical clearance to conduct this study was obtained from the Armauer Hansen Research Institute/ALERT Ethics Review Committee (PO/08/210). All research methods were performed in accordance with the Declaration of Helsinki. Potential respondents were invited to provide e-consent attached to the online questionnaire. To keep the confidentiality of respondents, the personal identifiers of the respondents and their RECs were coded.

## Results

### Socio-demographic characteristics of research ethics committees

**FIGURE 1:**
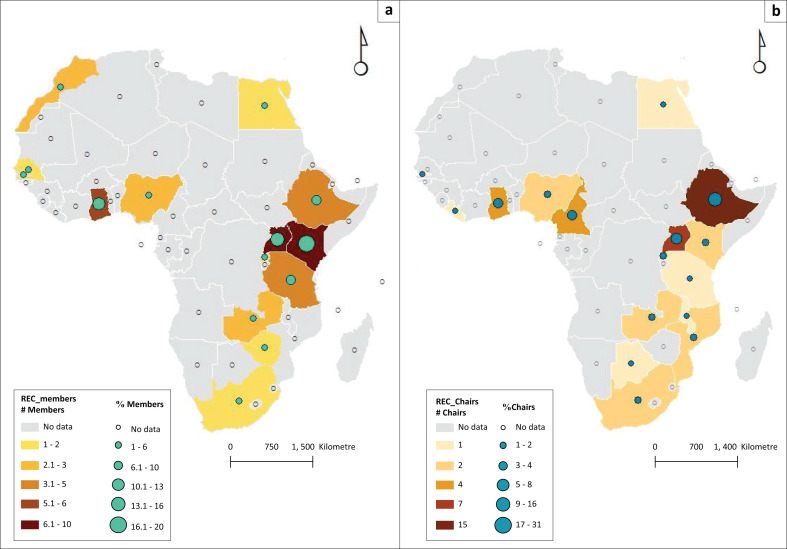
(a) Geographic distribution of research ethics committee members. (b) Geographic distribution of research ethics committee chairs.

In this survey, 600 emails were sent to REC chairs, 200 were delivered, and 400 emails came back with a failed delivery message. Out of the 200 emails, 98 (49%) responses were received, out of which 50 were REC members, while 48 were REC chairs. Out of the total, only three responded in French. Study participants were from 16 African countries, and they were from the North Africa (Egypt), Southern Africa (South Africa, Botswana, Mozambique, Malawi and Zambia) and Eastern African (Ethiopia, Kenya, Uganda, Rwanda and Tanzania) and Western African region (Ghana, Nigeria, Gambia, Liberia and Cameroon) (Table 1 and Table 2, Figure 1). The mean (standard deviation [s.d.]) age of REC members was mean 47 (s.d. ±12) years, and over half of them were female, 27 (54%). The majority of REC members and chairs were physicians; however, nearly 20% of the members are not represented by a layperson in the committee. Over three-fourths of REC chairs reported that the number of REC members ranges from 9 to 20. Almost half of REC members’ affiliations were outside the REC’s home institution (Table 1 and Table 2).

**TABLE 1:** Socio-demographic characteristics of research ethics committee members.

REC members	*n*	%
**Region and country**		
East Africa	29	58
West Africa	12	24
Southern Africa	5	10
North Africa	4	8
**REC members role or profession**		
Physician	13	26
Other medical practitioners†	3	6
Layperson/community representative	3	6
Biomedical scientist	8	16
Social scientist	7	14
REC secretary	11	22
Ethicist	7	14
Others	8	16
Being a member of a professional ethics organisation	26	53

REC, research ethics committee.

† , Other medical practitioners: Nurses, pharmacists, laboratory technologies, etc.

**TABLE 2:** Socio-demographic characteristics of research ethics committee chairs.

REC chairs	*n*	%
**Region and country**		
East Africa	27	56
West Africa	12	25
Southern Africa	8	17
North Africa	1	2
**REC members role or profession**		
Physician	41	85
Other medical practitioner	38	79
Biomedical scientist	37	77
Social scientist	36	80
Lawyer	23	51
Lay person or community representative	37	82
Ethicist	24	50
Other†	12	25
REC members are officially employed at REC home base institution (quantitative) mean %, s.d.	52	32
< 5 members	11	34
6–10 members	13	41
> 10 members	8	26
REC members are not officially employed at REC home base institution, mean %, s.d.	48	32
< 5 members	24	53
6–10 members	10	22
> 10 members	6	13

REC, research ethics committee; s.d., standard deviation.

† , Other medical practitioners: Nurses, pharmacists, laboratory technologies, etc.

### Research ethics committee characteristics and working documents

Nearly half (49%, *n* = 22) of RECs were academic institute affiliated. All of them reported having different types of SOP and forms; most of them have SOP on initial (98%, *n* = 49) and post-approval review (92%, *n* = 46), but only 76% (*n* = 38) have SOP on serious adverse event (SAE) review. Seventy per cent of REC members reported having regular monthly meetings.

Ninety per cent (*n* = 43) of REC chairs claimed that their REC is accredited; however, only 35 (70%) of them provided the name of the organisation providing accreditation. Half of the accredited RECs (52%, *n* = 22) reported being recognised by a ‘National Regulatory Authority’ in their respective country. Only three chairs reported the recognition of their RECs by SIDCER, all based in East Africa, and one REC reported being accredited by the Association for the Accreditation of Human Research Protection Programs (AAHRPP).

Over 80% (*n* = 43) of REC members reported reviewing different types of research protocols, including clinical, laboratory, public health and socio-behavioural research. Only 58% (*n* = 29) of RECs practiced exemption of protocols from ethics review when appropriate, while 94% (*n* = 47) did an expedited review of protocols when the need arose.

Most (91%, *n* = 42) REC members responded to their involvement as primary reviewers for scientific and technical issues (Table 3). The majority of REC members (82%, *n* = 41) and chairs (94%, *n* = 45) reported that they have reviewed COVID-19-related protocols. The most common protocols reviewed were public health interventions and community engagement. On the flip side, there were relatively few protocols for new drug tests, drug repurposing and diagnostic tools.

**TABLE 3:** Research ethics committee characteristics, activities and working documents.

REC affiliation	*n*	%
**REC members**		
Academic Institute	21	42
Hospital (public or private)	12	24
Research institute	17	34
Independent	3	6
Other	5	10
**Items in the standard operating procedure**		
List of international or national guidelines and regulations	37	74
Structure and composition of the REC	48	96
Initial review procedures	49	98
Post-approval review (amendments, progress & final reports),	46	92
Review of SAE reports	38	76
Review of protocol deviation or violation reports	41	82
Agenda and minutes preparation,	42	84
Documentation of protocol files	44	88
Archiving	42	84
Standard researcher application form	47	94
Standard assessment form of the protocol and informed consent	42	84
Others	4	8
**Reviewing the following types of protocols**		
Clinical research	43	86
Socio-behavioural research	43	86
Laboratory research	39	78
Public health research	43	86
Others	2	4
Did not review	3	6
**Types of review by REC**		
Exempt	29	58
Expedited review	47	94
Full board review	46	92
Joint or centralised review of multisite protocols	18	36
**REC meeting frequency**		
Weekly or fortnight	5	10
Monthly	35	70
Quarterly (every 3 months)	7	14
Semester (every 6 months) or annually	3	6
**Review functions**		
Primary reviewer for scientific/technical issues,	42	91
Primary reviewer for informed consent	29	63
Others	9	2
**REC chairs**		
Academic institute	22	49
Hospital (public or private)	9	19
Research institute	10	21
Independent	9	19
Other	8	18
**List of international or national guidelines and regulations**		
Declaration of Helsinki	41	95
Council of the International Organization for Medical Sciences	28	65
International Conference for the Harmonization of GCP	35	81
WHO Ethics Guidelines	36	84
Local guideline regulations	33	77
Other	5	12
Accredited or recognised (yes)	43	90
**If yes, who accredited (*n* = 43)**		
National Regulatory Authority	22	52
SIDCER	3	7
AAHRPP	1	2

AAHRPP, The Association for the Accreditation of Human Research Protection Programs; SIDCER, Strategic Initiative for Developing Capacity in Ethics Review; REC, research ethics committee; GCP, Good Clinical Practice; SAE, serious adverse event; WHO, World Health Organization.

### Activities and demands of research ethics committees during the COVID-19 pandemic

REC chairs who participated in the survey reported that their REC reviewed 5860 protocols in 2019, while in 2020 the number increased by 744 (12.7%). In both years, over half of RECs reviewed less than 100 protocols each. As per the REC members’ report, there was an increment in protocol amendment (79%, *n* = 38), deviation/violation (29%, *n* = 14) and early protocol termination (25%, *n* = 12). In reviewing COVID-19 protocols, the most common challenges faced by REC members/chairs were issues related to risk–benefit assessment, scientific design and informed consent.

Institutional policies for the prevention of COVID-19, such as limiting face-to-face meetings (96%, *n* = 46), limits on access to facilities (69%, *n* = 33), limits in the number of people in a room (81%, *n* = 39) and 71% (*n* = 34) of REC limits travel. Accordingly, during the pandemic, RECs have shifted their meetings to either virtual platforms (47%, *n* = 23) or hybrid meetings (49%, *n* = 24), (Table 4).

**TABLE 4:** Research ethics committee activities and demands during the COVID-19 pandemic.

REC members	*n*	%
Participated in review of COVID-19-related protocols	39	81
**Elements reviewed by REC for COVID-19 protocol**		
Qualifications of the investigator	36	84
Conflicts of interest	30	70
Scientific soundness	37	86
Informed consent	39	91
Advertisement/recruitment materials	23	54
Vulnerability	37	86
Risks	41	95
Benefits	40	93
Privacy	36	84
Others	4	9
**Difficult issues to review**		
Risks/benefits	16	36
Conflict of interest	9	21
Privacy issues	6	14
Vulnerability	7	16
Scientific design	11	25
No difficulty	16	36
Others	2	4
Need of training in ethics review	40	80
**Training topics in ethics review interested to attend**		
Principles of research ethics	14	31
International guidelines and local regulations	22	49
Review of clinical research	25	56
Review of socio-behavioural research	22	49
Community engagement research	26	58
Types of consent forms	17	37
Vulnerability	20	44
Risk–benefit assessment	25	56
Not interested for training	4	9
Others	2	4
**REC chair**		
REC reviewed COVID-19 or related protocols	45	94
**Types of COVID-19 protocol reviewed**		
Dx. tool for COVID-19	24	53
New drug for COVID-19	13	27
Old drug tested for COVID-19	15	31
Vaccine for COVID-19	16	33
Convalescent plasma for COVID-19	5	10
Public health intervention for COVID-19	39	81
Community engagement for COVID-19	31	65
Other	5	10
**Issues challenging during COVID-19**		
Scientific design	23	48
Informed consent	24	50
Conflict of interest	8	17
Vulnerability	12	25
Risks	30	63
Benefits	10	21
Privacy confidentiality	20	42
**Recommended training for REC members**		
Online review meetings	36	75
Ethical issues COVID-19 clinical trials	36	75
Ethical issues COVID-19 vaccine trials	36	75
Ethical issues COVID-19 PH research	37	77
Ethical issues COVID-19 socio-behavioural research	25	52
Types informed consent	29	60
Other	6	13
New protocol submissions reviewed in 2019 (sum)	5860	-
< 100 per respondent (*n* = 1032)	29	64
101–200 per respondent (*n* = 1332)	9	20
201–500 per respondent (*n* = 970)	3	7
≥ 500 per respondent (*n* = 2526)	4	9
New protocol submissions reviewed in 2020 (Sum)	6604	-
< 100 (*n* = 889)	23	52
101–200 (*n* = 1843)	15	34
201–500 (*n* = 1377)	4	9
≥ 500 (*n* = 1436)	2	5
COVID-19 cases	-	-
< 10 000	9	20
> 100 000	21	47
10 001 to 100 000	15	33
Adopted COVID-19 policies	-	-
Limit face to face	46	96
Limit access institutional facility	33	69
Limit no of people in a room	39	81
Limit travel	34	71
Other	6	13
Mode of meeting	-	-
Face to face	2	4
Online	23	48
Both/hybrid	24	50
**COVID-19 impact on number of protocols**	-	-
Increases	20	42
Decreases	15	31
No effect	13	27
Protocol deviation/violation (yes)	14	29
Amendments	38	79
Early termination of protocol	12	25
Serious adverse events	11	23
Subject complaints	7	15

PH, Public Health; Dx, Diagnosis; REC, research ethics committee.

### Knowledge, attitudes and training needs among research ethics committees

The majority of REC members (*n* = 40) recommended training in specific topics, including risk–benefit assessment, community engagement, clinical and socio-behavioural research and various types of guidelines and regulations. Similarly, REC chairs identified the need for training on the ethical issues of clinical trials, public health research and how to conduct online review meetings (Table 5 and Table 6).

**TABLE 5:** Knowledge of research ethics committee member towards COVID-19 protocol review.

REC knowledge towards COVID-19 reviewing protocols	Correct answer	
	*n*	%
RECs should review for approval unproven drugs vaccines and intervention	8	17
Participant consent should be required for COVID-19 clinical trial protocol	49	98
COVID-19 patients enrolled in clinical trials are vulnerable	40	80
Healthy volunteers enrolled in vaccine trials are vulnerable	36	72
COVID-19 patients in hospitals should not be allowed to join a clinical trial	32	64
Traditional medicine for COVID-19 therapy should undergo a clinical trial	42	84
Only patients should sign the consent forms for COVID-19 research	25	50
A drug company may appoint an employee to become the principal investigator	35	70
REC members should sign a conflict of interest and confidentiality declaration	47	94
The identity of COVID-19 participants in research should be kept confidential	49	98

REC, research ethics committee.

**TABLE 6:** Attitude of research ethics committee member towards COVID-19 protocol review.

RECs attitude towards COVID-19 reviewing protocols	(strongly) Disagree		Neutral		(strongly) Agree	
	*n*	%	*n*	%	*n*	%
RECs need training in ethics review of COVID-19 protocols	3	6	3	6	44	88
Multicentre COVID-19 protocols should be reviewed by joint centralised RECs	4	8	5	10	41	82
Joint central RECs ensure harmonised recommendations	2	4	5	10	42	86
All COVID-19 protocols are high-risk protocols	22	44	13	26	15	30
Any medical doctor can be appointed as principal investigator	43	86	5	10	2	4
Any medical doctor can review the technical issues	36	75	7	14	7	14

REC, research ethics committee.

With regard to the knowledge of REC members towards the COVID-19 protocol review, only four members responded that REC has no mandate on new drug or vaccine approval. On other knowledge questions, REC members scored from 60% to 98% (Table 5). Forty-four per cent of respondents (*n* = 22) did not believe that all COVID-19-related proposals are high-risk protocols. Only a few agree that physicians can be the principal investigator (4%, *n* = 2) or reviewers of COVID-19 protocol (14%, *n* = 7) (Table 5).

## Discussion

To the best of our knowledge, this was the first study conducted in Africa assessing RECs activities and the impact of the COVID-19 pandemic on RECs functioning. Sixteen African countries’ RECs participated in this study. Most of them were from eastern African nations, and participants from only one country reported having SIDCER recognised RECs. Almost all REC members reported being involved in REC activities, including COVID-19 protocol review during the pandemic with virtual and/or hybrid meeting platforms. The most important COVID-19 pandemic-induced challenges include assessing risk–benefit and ethical issues unique to the pandemic and RECs recommended training in those areas.

The WHO highlights important considerations regarding the composition and expertise of RECs. The presence of a layperson as a requirement for the quorum of the board meeting is indeed outlined in the standards.^18^ This requirement aims to ensure that the perspectives and interests of the general public, who may not have specialised scientific or medical knowledge, are represented in the decision-making process. However, studies showed that not all RECs have a layperson in their members composition,^19,20^ indicating a potential gap in meeting this requirement. Additionally, it has been observed that some RECs lack the relevant professional compositions, such as biomedical scientists, social scientists and ethicists, who can provide valuable expertise in evaluating research protocols from different perspectives.

The primary role of RECs is to protect the dignity, rights, safety and well-being of research participants.^21^ Inadequate representation of experts in the committee may lead to an adverse outcome for patients. The absence of diverse expertise can result in the overestimation of benefits, the underestimation of harm and a lack of timely awareness regarding potential benefits or harm associated with the research.^22^ To comply with standards and guidelines, RECs must include the relevant expertise and ensure the presence of a layperson in their committees. This would help foster comprehensive evaluations of research protocols, promote a balanced consideration of scientific, ethical and societal aspects and ultimately enhance the protection of research participants’ rights and welfare.

In this study, the majority of regular members in RECs are physicians, which aligns with the number of clinical studies being reviewed.^23,24^ This observation suggests that physicians often play a significant role in RECs, given their expertise and involvement in clinical research.

It is recommended that the RECs be established with members drawn from both external and local institutions to ensure balanced representation and independence. This diverse composition helps demonstrate the independent nature of the RECs^18^ and ensures a broad range of perspectives in the decision-making process. The study’s finding that almost half of the REC members were non-affiliated with REC’s home base institutions suggests a degree of independence in the composition of the committees.^18^

While there is no established gold standard for the number of REC members, it is generally recognised that the number should be based on the volume of workload and the size of the REC’s home base institution. The study found that the majority of REC chairs reported having nine to 20 members, which falls within the acceptable range and indicates a reasonably sized committee for effective decision-making and review processes.^18^

It is worth noting that most RECs in the study met regularly every month and made use of the primary review process. The primary reviewer system is a procedure commonly used in various research committees globally.^20,25^ The purpose of this system is to ensure that protocols submitted for review are assigned to members who possess the relevant expertise and knowledge in the specific area of research. Reviewers with knowledge and experience in the specific area can assess the scientific validity, ethical considerations and potential risks and benefits associated with the research. By employing the primary reviewer system, RECs aim to ensure comprehensive evaluation of research protocols by the assigned reviewer(s) and combining the expertise of multiple members. This helps to maintain scientific rigour, protect the rights and welfare of participants and uphold ethical standards in research.

All RECs claimed to have SOPs, documents and forms. In addition, REC chairs reported using various types of national and international guidelines for protocol review. However, all but three were confirmed to have local recognition authority, and only three had international recognition. This might be because of inconsistencies in regional standards or RECs’ self-perception of compliance with regional and international standards. Such a difference may compromise the quality, transparency and accountability of the protocol review process. International or regional recognition programmes help to standardise and disseminate best practices across the region. Therefore, we recommend such programmes reach out to all Africa-based RECs.

COVID-19 disrupted several activities, including research and protocol review.^6^ Despite the effect of the pandemic, the world struggled to tackle the virus, which ranged from public health interventions to vaccine development. Amidst this challenging period, RECs were forced to review protocols with speed in response to facilitate the urgent need for generating evidence-based practice and tackling the pandemic. According to our study, RECs reviewed over five thousand protocols in 2020, which was 13% higher than protocol reviews before the pandemic. The most common type of protocols reviewed was public health interventions for COVID-19 and community engagement. As previous reports showed that big pharmaceutical companies are based in developed countries and relatively, and there are relatively few clinical trials in Africa, which is also reflected in this study.^12,26^ Because of the poorly characterised nature of the coronavirus, case definition, prevention, diagnostic tools and variant nature resulted in continuous changes in protocol designs and guidelines.^27,28^ As indicated by Reyes et al., we also noted an increase in protocol deviation and amendments in protocols submitted to African RECs.^6^ The continuous changes in protocol design and introduction of new designs have added new challenges in the review process, and this has also been reflected in our findings, where the main challenges faced by RECs were risk–benefit assessment of COVID-19 protocols.

Studies where the likelihood of discomfort, harm or inconvenience to participants is not expected to exceed that encountered in daily life or while undergoing routine examinations or tests shall be exempted from review.^29^ This study revealed that the exemption of protocols was less practiced in Africa. The practice of exemption minimises workload and avoids the unnecessary delay of the review process for low-risk studies potentially deemed for further review. This may also be explained by low awareness, the absence of clear national guidelines or the risk-averse approach of RECs because of perceived risks. Accordingly, experience-sharing among RECs could promote the appropriate and consistent use of protocol exemptions.

There are knowledge gaps that were identified in the current study, which will be important for designing future need-based training. Important findings of this study were knowledge and attitude gaps towards COVID-19 protocol review, including RECs mandate on new drug/vaccine approval, and patient enrolment to COVID-19 clinical trials. Future training should also include training on community engagement and risk–benefit assessment. Despite the limited number of clinical trials available in Africa, there is a need for capacity-building packages in this area for RECs at our study sites.

The paramount implication derived from this study underscores the imperative to fortify RECs in Africa through diverse mechanisms. A pivotal intervention could involve the establishment of a regional REC platform and database, facilitating the tracking of RECs across the continent. The challenges identified, particularly those related to capacity-building, can be effectively addressed through initiatives that have the potential to reach numerous RECs throughout Africa.

One strategic approach involves strengthening regional platforms, such as the PABIN, which could play a vital role in supporting the RECs in our study areas by offering capacity-building initiatives through various training programmes. Collaborating with recognised programmes such as SIDCER or similar initiatives further enhances the credibility and effectiveness of these capacity-building endeavours. This comprehensive strategy aims to elevate the capabilities and standards of RECs within the study areas and the countries involved, ensuring a robust ethical framework for research activities.

During this study, we encountered difficulties in contacting RECs because of the absence of official website and email addresses. This limitation highlights a broader challenge in communicating with these committees. Our experience demonstrates that establishing a regional platform and a centralised database could significantly improve accessibility, facilitate communication and help to increase their visibility. Therefore, we recommend the development of such a system to address these challenges and support better governance and oversight in the study areas.

### Limitations

This study does have some inherent limitations. Initially, being an online survey, the response rate was low. The survey’s Google Form link was distributed to REC chairs, who were then requested to share it with their respective members, making it challenging to calculate the response rate among REC members because of the unknown number of emails forwarded by REC chairs.

Despite our efforts, which included sending email reminders in five rounds to maximise responses, the reasons behind the low response rate remain inadequately explained. Globally, the pandemic led to restrictions on face-to-face meetings and limited office access, prompting a shift to virtual work. However, the challenges of online work in Africa, where Internet coverage is limited, may have contributed to difficulties for some REC members in accessing and responding to our online survey.

Another notable limitation pertains to the institutional structure of RECs in Africa. A significant number of these committees lack an official website or an updated contact address, posing a challenge in our attempts to reach out to as many REC chairs as possible. These limitations should be considered when interpreting the findings of this study.

## Conclusion

Notwithstanding the challenges imposed by the pandemic, the RECs in our study sites demonstrated resilience by actively engaging in virtual and/or hybrid meeting platforms, successfully reviewing numerous COVID-19 protocols. The surge in submitted proposals, while indicative of ongoing research activities, has augmented the workload for these committees, introducing fresh ethical dilemmas related to research design, risk–benefit assessment and the informed consent process. It’s noteworthy that only a limited number of RECs received recognition from SIDCER.

In light of these findings, this study underscores the imperative to strengthen the RECs in the study areas. Suggested measures include the establishment of a regional REC platform and database to foster collaboration and information sharing, as well as the provision of periodic training opportunities tailored to the evolving needs of the committees, preferably through online platforms. Such initiatives aim to enhance the capacity and effectiveness of RECs in our study areas, enabling them to better navigate the complexities of ethical review in a dynamic and challenging landscape.
